# Japanese Encephalitis Virus Transmitted Via Blood Transfusion, Hong Kong, China

**DOI:** 10.3201/eid2401.171297

**Published:** 2018-01

**Authors:** Vincent C.C. Cheng, Siddharth Sridhar, Shuk-Ching Wong, Sally C.Y. Wong, Jasper F.W. Chan, Cyril C.Y. Yip, Chi-Hung Chau, Timmy W.K. Au, Yu-Yan Hwang, Carol S.W. Yau, Janice Y.C. Lo, Cheuk-Kwong Lee, Kwok-Yung Yuen

**Affiliations:** University of Hong Kong, Hong Kong (V.C.C. Cheng, S. Sridhar, S.C.Y. Wong, J.F.W. Chan, C.C.Y. Yip, K.-Y. Yuen);; Queen Mary Hospital, Hong Kong (V.C.C. Cheng, S.-C. Wong, T.W.K. Au, Y.-Y. Hwang);; Grantham Hospital, Hong Kong (C.-H. Chau);; Centre for Health Protection, Hong Kong (C.S.W. Yau, J.Y.C. Lo);; Hong Kong Red Cross Blood Transfusion Service, Hong Kong (C.-K. Lee)

**Keywords:** viral encephalitis, Japanese encephalitis virus, blood transfusion, vector-borne infections, immunocompromised host, infection control, encephalitis, China, viruses, packed red blood cells, platelets, asymptomatic

## Abstract

Japanese encephalitis virus (JEV) is a mosquitoborne virus endemic to China and Southeast Asia that causes severe encephalitis in <1% of infected persons. Transmission of JEV via blood transfusion has not been reported. We report transmission of JEV via blood donation products from an asymptomatic viremic donor to 2 immunocompromised recipients. One recipient on high-dose immunosuppressive drugs received JEV-positive packed red blood cells after a double lung transplant; severe encephalitis and a poor clinical outcome resulted. JEV RNA was detected in serum, cerebrospinal fluid, and bronchoalveolar lavage fluid specimens. The second recipient had leukemia and received platelets after undergoing chemotherapy. This patient was asymptomatic; JEV infection was confirmed in this person by IgM seroconversion. This study illustrates that, consistent with other pathogenic flaviviruses, JEV can be transmitted via blood products. Targeted donor screening and pathogen reduction technologies could be used to prevent transfusion-transmitted JEV infection in highly JEV-endemic areas.

Japanese encephalitis virus (JEV) is a member of the genus *Flavivirus* and is the eponymous member of the JEV antigenic complex of viruses that also includes West Nile virus (WNV). JEV is endemic to Southeast Asia and China, where ≈67,900 cases occur every year (*1*). The virus is maintained in a complex enzootic cycle involving pigs and birds; humans are infected via the bite of infected *Culex* spp. mosquitoes (particularly *C. tritaeniorhynchus*) (*2*). Humans infected with JEV have low viremia levels and are considered to be dead-end hosts (*3*). Although neurologic manifestations are observed in <1% of infected patients, encephalitis caused by JEV is a devastating condition with a mortality rate of 20%–30%. Survivors often suffer permanent neurologic sequelae. No treatment has been proven to be effective for Japanese encephalitis in clinical trials (*4*).

Arthropodborne viruses are an emerging threat to the blood supply. Transmission via blood transfusion has been described for 4 arthropodborne viruses: dengue virus, WNV, tick-borne encephalitis virus, and Zika virus (*5**–**10*). Furthermore, chikungunya virus and Usutu virus have also been found in blood donor samples, indicating a risk for transmission via this route as well (*11**,**12*). In contrast, transfusion-related JEV transmission has not been reported in the literature, although the potential for this type of transmission has been recognized (*13*). In this study, we describe a case of nosocomial Japanese encephalitis in an immunocompromised lung transplant recipient. An outbreak investigation was conducted to ascertain the source of the infection and if other patients were at risk for Japanese encephalitis.

## Materials and Methods

### Setting

Queen Mary Hospital is a 1,700-bed, university-affiliated, tertiary referral center in Hong Kong that has a lung transplantation service. After lung transplantation, patients are transferred for extended care to Grantham Hospital, a 388-bed specialized respiratory care hospital in the healthcare network of Hong Kong West Cluster.

### Virologic Investigations

Virologic investigations for JEV were performed at the Public Health Laboratory Services Branch, Centre for Health Protection, of the Hong Kong Department of Health and the microbiology laboratory at Queen Mary Hospital. We performed JEV IgM testing on serum and cerebrospinal fluid (CSF) using the JE Detect IgM Antibody Capture ELISA (InBiOS, Seattle, WA, USA). We amplified flavivirus RNA in clinical specimens using a conventional panflavivirus heminested reverse transcription PCR (RT-PCR) with primers targeting the nonstructural protein 5 (NS5) gene as described previously (*14*). We detected RT-PCR products using gel electrophoresis after the first and second rounds of amplification. We performed real-time RT-PCR specific for JEV using an in-house‒developed assay with primers targeting the NS5 gene (forward primer 5′-GGAGCTGGATGGAATGTGAA-3′, reverse primer 5′-TCCCTCCGATGGAAGTATAGAA-3′, probe 6-FAM-CCAAAGCGTATGCACAGATGTGGC-BBQ-650; Technical Appendix 1). We tested for other pathogens that can cause encephalitis, including herpes simplex virus, varicella zoster virus, enterovirus, adenovirus, cytomegalovirus, erythroparvovirus B19, rabies virus, *Mycobacterium tuberculosis*, *Mycoplasma pneumoniae*, and *Toxoplasma gondii*, using in-house‒developed PCR and RT-PCR assays (*15*).

### Sequencing and Phylogenetic Analysis

Using the inner primer pairs of the panflavivirus heminested RT-PCR, we performed Sanger sequencing of flavivirus NS5 gene amplicons with the ABI PRISM 3130xl Genetic Analyzer (Applied Biosystems, Foster City, CA, USA). We compared the resulting sequences to the sequences in GenBank using BLAST (*16*) and performed phylogenetic analysis using MEGA 6.06 (http://www.megasoftware.net/). We performed multiple alignment with the NS5 gene sequences obtained from this study (167 nt long) and those of other JEV strains using ClustalW, followed by phylogenetic tree construction using a Kimura 2-parameter substitution model plus invariant site and the maximum-likelihood method as previously described (*17**–**19*).

### Case Definitions

Patients were defined as having JEV infection if any 1 of 2 laboratory criteria was met: detection of JEV-specific IgM in CSF or serum specimens, or detection of JEV RNA in blood or CSF specimens. Patients were defined as having a confirmed transfusion-transmitted JEV infection if they met the above laboratory criteria for JEV infection and received a blood product transfusion from a donor with JEV viremia during the 3 weeks before illness onset. We performed phylogenetic analysis to compare the blood donor and recipient JEV sequences whenever sequences were available.

### Outbreak Investigation

Clinical details of the index patient, including date of transplantation, dates of blood transfusions, immunosuppressant dosages, and laboratory investigation results, were retrieved from the electronic patient record system. Archived clinical specimens from the index patient, including serum, CSF, bronchoalveolar lavage fluid, feces, urine, and saliva samples, were retrieved from the microbiology laboratory at Queen Mary Hospital and tested using the panflavivirus heminested PCR assay.

We retrieved details on the organ donor and other transplant recipients from the organ donor registry. We retrieved the remaining blood products of persons who donated blood that was transfused into the index patient during the 3 weeks before illness onset from the Hong Kong Red Cross Blood Transfusion Service and tested these samples using the panflavivirus RT-PCR and JEV-specific RT-PCR described previously.

Because the index patient had resided in Grantham Hospital for the entire JEV incubation period, mosquito presence in Grantham Hospital was directly assessed by interviewing healthcare workers, as described in our previous outbreak investigations (*20**–**23*). *Culex* vector surveillance is performed by the Food and Environmental Hygiene Department in selected areas of Hong Kong. We were able to retrieve the monthly surveillance data from 1 of their surveillance sites near Grantham Hospital (2.5 km away). This study was approved by the institutional review board of the University of Hong Kong and Hospital Authority Hong Kong West Cluster.

## Results

### Index Patient

**Figure 1:**
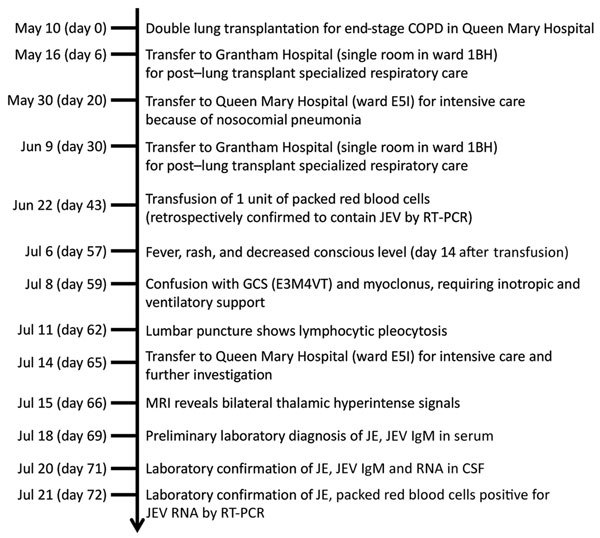
Timeline of index patient with transfusion-transmitted JEV infection, Hong Kong, China, May‒July 2017. Day counts indicate the number of days after double lung transplant, unless specified otherwise. COPD, chronic obstructive pulmonary disease; CSF, cerebrospinal fluid; GCS, Glasgow Coma Scale; JEV, Japanese encephalitis virus; MRI, magnetic resonance imaging; RT-PCR, reverse transcription PCR.

A 52-year-old man with advanced chronic obstructive pulmonary disease underwent double lung transplantation on May 10, 2017, in Queen Mary Hospital. After transplantation, the patient’s clinical course was complicated by nosocomial pneumonia; *Burkholderia cepacia* and methicillin-resistant *Staphylococcus aureus* were isolated from the patient’s sputum specimens. The infection required a prolonged course of broad-spectrum antimicrobial drugs and ventilator support. Antirejection prophylaxis included tacrolimus (0.5 mg/d), mycophenolate mofetil (250 mg 2×/d), and a tapering course of prednisolone. He was transferred multiple times between Queen Mary Hospital and Grantham Hospital (Figure 1). The patient required transfusions of packed red blood cells for anemia on June 20, 22, and 25 (1 unit/d) in Grantham Hospital.

On July 6 (57 days after transplantation and 11‒16 days after transfusions), he had a transient maculopapular rash and fever. Blood tests showed leukocyte (8.99 × 10^9^ cells/L), neutrophil (7.17 × 10^9^ cells/L), and lymphocyte (1.18 × 10^9^ cells/L) counts within reference ranges. Plasma sodium decreased to 128 mmol/L, and liver and renal function test results were unremarkable. His conscious level progressively deteriorated, and he developed myoclonic jerks 2 days after the onset of rash. Serial blood tests showed transient lymphopenia (0.44 × 10^9^ cells/L) and a drop in platelet count (33 × 10^9^/L).

**Figure 2:**
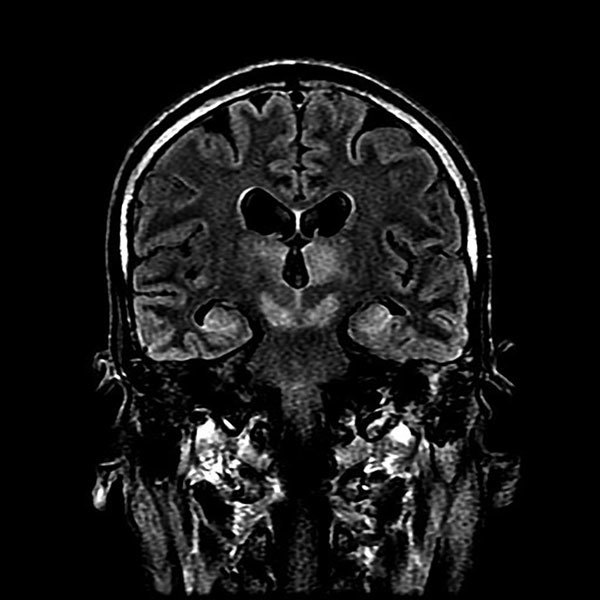
Magnetic resonance imaging of brain of index patient 66 days after double lung transplantation, Hong Kong, China. Coronal FLAIR (FLuid Attenuation Inversion Recovery sequence) image of the head at the level of the lateral ventricles, thalamus, and midbrain shows high signal at bilateral thalamus, midbrain, and medial temporal lobes.

A lumbar puncture was performed 6 days after symptom onset. The opening pressure was 13.4 cm H_2_O (reference range 6‒20 cm H_2_O), and the CSF had a total cell count of 20 × 10^6^ cells/L, with a predominance of monocytes (58%) and lymphocytes (35%). CSF protein was elevated (1.61 g/L). Magnetic resonance imaging (MRI) of the brain performed 10 days after symptom onset showed symmetric hyperintensities in the bilateral thalami, substantia nigra, and medial temporal lobes (Figure 2).

In view of the CSF pleocytosis and elevated protein levels, an extensive workup was performed for infectious causes of meningoencephalitis. Gram and Ziehl-Neelsen stains, bacterial and fungal cultures, and the cryptococcal antigen test all gave negative results. PCR for herpes simplex virus, varicella zoster virus, cytomegalovirus, Epstein-Barr virus, enterovirus, rabies virus, *Mycobacterium tuberculosis*, *Mycoplasma pneumoniae*, and *Toxoplasma gondii* were all negative. Serum PCR screening for adenovirus and erythroparvovirus B19 were also negative.

Because of the characteristic distribution of MRI abnormalities involving the basal ganglia, the possibility of flaviviral encephalitis was considered. The patient’s CSF was positive for JEV IgM. Subsequent serum samples collected July 15 and 18 were also positive for JEV IgM, but the archived serum specimen obtained May 31, 2017, was negative (Table). Panflavivirus heminested PCR performed on CSF yielded a positive result, with sequencing of the amplicon confirming the presence of JEV (genotype 1). Archived clinical specimens obtained from the patient during the 2 weeks before symptom onset were retrospectively tested using the panflavivirus heminested RT-PCR. Two serum specimens collected June 26 and 28 (10 and 8 days, respectively, before symptom onset) and a bronchoalveolar lavage fluid specimen collected day 5 after symptom onset also tested positive for flavivirus RNA and were confirmed to be JEV by sequencing (GenBank accession no. MF594404). The patient died on October 1, 2017.

**Table:** Demographics, investigation results, and clinical details of asymptomatic JEV-infected blood donor and recipients, Hong Kong, China, May‒July 2017*

Variable	Donor	Packed red blood cell recipient (index patient)	Platelet recipient	Plasma recipient
Underlying disease	No history of disease, no recent travel to JEV-endemic regions outside Hong Kong; resides in JEV-endemic area Tin Shui Wai, Yuen Long	End-stage chronic obstructive pulmonary disease with lung transplantation, May 10	Acute myeloid leukemia post induction chemotherapy with cytarabine and daunorubicin	Intracranial hemorrhage
Blood donation or transfusion type, date	Blood donation, May 29	Packed red blood cell transfusion, June 22	Platelet transfusion, June 2	Plasma transfusion, June 20
Pretransfusion IgM serology result, date	Specimen not available	Serum negative, May 31	Serum negative, May 25	Specimen not available
Posttransfusion IgM serology result, date	Serum positive, July 22	CSF positive, July 11; serum positive, July 15 and 18	Serum positive, July 5 and 22	Specimen not available
JEV nucleic acid test, specimen type and result, date	Positive archived blood specimen	Positive blood sample, June 26 and 28; positive bronchoalveolar lavage, July 10	Negative plasma sample, July 22; negative urine sample, July 25	Specimen not available
Clinical symptoms	Asymptomatic	Fever and rash July 6 (14 d after transfusion), followed by decreased consciousness and myoclonic jerks; MRI showed typical appearance of T2 hyperintensity of bilateral thalami, substantia nigra, and medial temporal lobes	Asymptomatic	Unknown
Outcome	Full recovery	Died October 1, 2017	Full recovery	Died from respiratory failure 14 d after transfusion

*CSF, cerebrospinal fluid; JEV, Japanese encephalitis virus; MRI, magnetic resonance imaging.

### Outbreak Investigation

In response to this case of nosocomial acquisition of JEV, the hospital infection control team launched an outbreak investigation in Queen Mary Hospital and Grantham Hospital by tracing the placement of the patient during his entire hospitalization. The index patient was treated in air-conditioned rooms in Queen Mary Hospital and Grantham Hospital throughout his hospitalization. Therefore, mosquitoborne transmission was considered unlikely. Furthermore, a vector surveillance point surveyed by the Food and Environmental Hygiene Department, located 2.5 km from Grantham Hospital, showed that *C. tritaeniorhynchus* mosquitoes had not been detected since April 2017. Therefore, alternative sources of infection, including blood transfusion, sharps injury with contaminated blood, and organ transplantation, were considered.

The onset of symptoms in the index patient was 57 days after the transplantation date, longer than the usual 5‒15-day incubation period for JEV. The organ donor was a 70-year-old road traffic accident victim. Urgent contact tracing of the recipients who received organs (1 liver and 2 kidneys) from this donor revealed that the recipients were asymptomatic. JEV IgM testing of serum samples from these transplant recipients on day 71 (for the liver transplant recipient), day 76 (for renal transplant recipient 1), and day 83 (for renal transplant recipient 2) after transplantation were all negative. A serum sample from the organ donor was not available for JEV testing.

**Figure 3:**
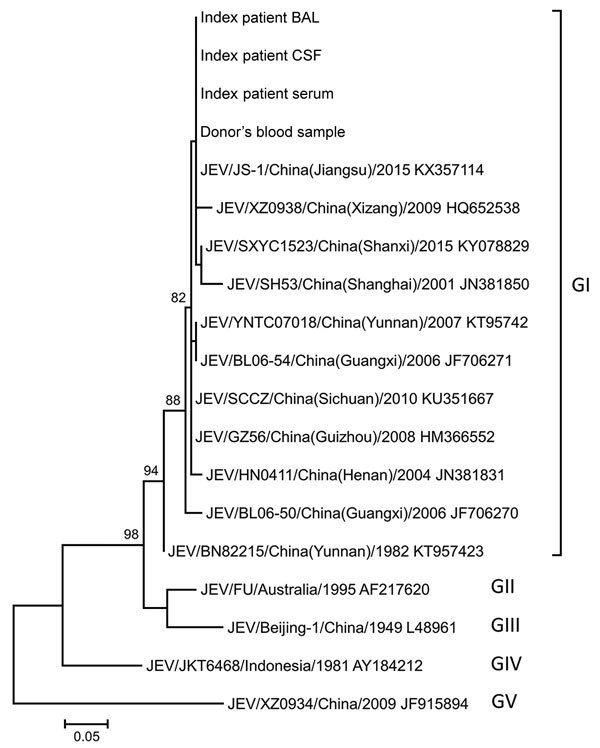
Phylogenetic tree constructed by using partial nonstructural protein 5 (NS5) sequences of JEV isolates detected in index patient and donor blood samples, Hong Kong, China, and other JEV reference strains available in GenBank (accession numbers shown). The tree was inferred from data by using the maximum-likelihood method with bootstrap values calculated from 1,000 trees. Only bootstrap values >70% are shown. A 167-nt fragment of NS5 from each virus was used in this analysis. Labels at right indicate JEV genotypes (GI–V): JEV from patient and donor samples grouped with GI strains. Scale bar indicates estimated number of nucleotide substitutions per 20 nt. BAL, bronchoalveolar lavage; CSF, cerebrospinal fluid; JEV, Japanese encephalitis virus.

Because the index patient had received 3 units of packed red blood cells from 3 different donors on 3 different days during the 3 weeks before illness onset (i.e., 1 unit each on June 20, June 22, and June 25), the remaining blood products from all 3 donors were traced from the Hong Kong Red Cross Blood Transfusion Service and samples were taken. Only the sample from the transfusion on June 22 tested positive by both real-time RT-PCR for JEV and conventional heminested PCR for flavivirus. Sequencing of the amplicons obtained by conventional PCR yielded a sequence identical to that obtained from the index patient (Figure 3). Phylogenetic comparison with other JEV sequences from GenBank showed that the index case and blood donor isolates belonged to JEV genotype 1, a circulating genotype common in southern China (*24*). The blood donor was a 46-year-old man who resided in Tin Shui Wai of the Yuen Long District in the New Territories region of Hong Kong. He had been asymptomatic at the time of blood donation (May 29, 2017) and had no recent travel to JEV-endemic regions outside of Hong Kong. He did not develop any symptoms of Japanese encephalitis after the blood donation. On July 22, a serum sample from the donor tested positive for JEV IgM. He was deferred from donating blood for 1 year.

### Evaluation for Secondary Cases

After confirmation that a blood donor was the source of JEV infection in the index patient, we urgently traced other patients who received blood products from this donor and identified 2 recipients (Table). One was a 61-year-old man with newly diagnosed acute myeloid leukemia who had received induction chemotherapy with cytarabine and daunorubicin during May 8–14, 2017; he was transfused with platelets obtained from the viremic blood donor on June 2, 2017. His white blood cell count on the day of platelet transfusion was 3.39 × 10^9^ cells/L, and he had severe lymphopenia (0.24 × 10^9^ cells/L). His lymphocyte counts steadily rose to 0.95 × 10^9^ cells/L 10 days after the transfusion and normalized by the end of June. He was asymptomatic at follow-up 2 months after transfusion. Upon recognition that this patient received a potentially JEV-infected blood product, staff retrieved his archived serum specimens collected before transfusion and 33 and 50 days after transfusion to test for JEV IgM. The serum specimens collected before transfusion tested negative for JEV IgM, but those collected after tested positive, confirming a recent asymptomatic infection probably contracted from the platelet transfusion. JEV RNA was not detected in a urine specimen collected 50 days after or a plasma sample collected 53 days after the transfusion.

The other blood product recipient was a 64-year-old man who was admitted for intracranial hemorrhage. He received a plasma transfusion June 20, 2017, and died July 4, 2017, due to respiratory failure. No serum specimens were available for serologic testing from this patient, and an autopsy was not performed.

## Discussion

In our study, we report nosocomial transmission of JEV through blood transfusion products from an asymptomatic viremic donor to 2 immunocompromised persons, resulting in 1 case of severe encephalitis and another asymptomatic infection with seroconversion. JEV has been documented to be exclusively transmitted via the bite of *Culex* mosquitoes. However, when we diagnosed JEV infection in a patient who had been hospitalized for a prolonged period of time, mosquitoborne transmission was considered unlikely because of several factors. First, the index patient was treated in an air-conditioned facility with closed doors and windows during the entire incubation period. Second, rigorous integrated pest management programs (including weekly inspections by dedicated staff and regular monthly pest control services) were implemented in public hospitals and clinics in Hong Kong in 2014 in response to the increasing threat of mosquitoborne infectious diseases, such as dengue in nearby Guangzhou Province, China (*25*). Furthermore, although real-time information on *Culex* spp. breeding density in the district was not available, a *C. tritaeniorhynchus* mosquito distribution survey in Hong Kong showed that adult vector prevalence was low in the Aberdeen area, where Grantham Hospital is located (*26*).

Because of these circumstances, other sources of infection were considered. Transmission via organ transplantation, which has been reported for WNV, was considered (*27*), but serologic screening of other organ recipients from the same donor did not reveal any evidence of JEV infection. Blood product transfusion was then considered as a possible source of infection. JEV RNA was detected in a blood donation sample from 1 donor, and phylogenetic analysis showed 100% sequence identity between the isolates in the donor and recipient, confirming transfusion-transmitted JEV.

The blood donor resided in Tin Shui Wai of the Yuen Long District, an area that has been shown to have a high *C. tritaeniorhynchus* vector density (*26*). From January 2003 through June 2017, seventeen cases of locally acquired JEV infection in Hong Kong were recorded by the Department of Health, and 11 (64.7%) of the 17 patients resided in Yuen Long District. Therefore, the donor was likely to have been infected in Tin Shui Wai. This report illustrates that localized pockets of high JEV endemicity can facilitate the transmission of JEV via unconventional routes.

Arboviruses pose unique threats to the blood supply (*28**–**30*). Compromise of the blood supply by arboviruses has been well documented during explosive outbreaks of Zika, dengue, WNV, and chikungunya virus infections in recent years (*11**,**31*). Our findings have major implications for JEV-endemic areas, where undetected transmission of JEV by blood transfusion might be widespread because of high rates of asymptomatic infection in both donors and recipients. In the case we report, 24 days elapsed between the time of blood donation and transfusion to the index patient, indicating that the virus can remain viable in packed red blood cells over a prolonged period of time. Risk assessment of the presence of JEV in the blood supply in JEV-endemic areas should be undertaken.

Mitigation of such transmission is difficult because standard measures to protect the blood supply during arboviral outbreaks, such as donor symptom and travel questionnaires (Technical Appendix 2), are not useful in the case of JEV, which has a high rate of subclinical infection and low incidence in Hong Kong. Two approaches to minimize the risk for transfusion-transmitted JEV infection are donor JEV screening and pathogen reduction technology (PRT). Both approaches have limitations. Donor screening would involve nucleic acid testing (NAT) of either individual donors or multiple donors by using minipools, as has been described for WNV and Zika virus (*32**–**34*). The biggest challenge for implementing screening is the lack of well-established and licensed JEV NAT for use in the blood donor setting. The use of clinical diagnostic NAT for donation screening is associated with the potential for false-positive results by cross-contamination caused by amplicon carryover. Individual donor NAT is likely to be prohibitively expensive, and minipool NAT is difficult to standardize for JEV, given the lack of data on viral loads in asymptomatic persons. Although historically most Japanese encephalitis cases in Hong Kong have occurred during the rainy summer season (May‒August), even limited seasonal screening of residents in high-risk areas of Hong Kong might be impractical because of the highly mobile population and imprecise delineation of seasons due to climate change. Although donor screening might not be cost-effective for universal application, a more selective application of blood products used for immunocompromised persons might be considered. However, JEV can cause life-threatening disease even in immunocompetent persons, and the correlation between immunosuppression and disease severity is not clear. A range of novel PRTs for blood products that involve the combined use of ultraviolet light and reagents such as psoralens or riboflavin are available (*35**,**36*). Examples include the INTERCEPT Blood System for platelets and plasma (Cerus Corporation, Concord, CA, USA); Mirasol Pathogen Reduction Technology for plasma, platelets, and whole blood (Terumo BCT, Lakewood, CO, USA); and the THERAFLEX platform for plasma and platelets (Macopharma, Tourcoing, France). These methods require individual component processing, are not suitable for all blood components, and are expensive to implement. No direct evidence indicates that these products are efficient at reducing JEV infectivity, although such efficacy could be extrapolated from studies on PRT for Zika and dengue viruses (*37**,**38*). Also, leukocyte depletion of blood products might theoretically reduce the risk for JEV transmission, but this method also requires validation. Implementation of a JEV vaccination program with high coverage in areas with high *C. tritaeniorhynchus* mosquito breeding density might also be considered to definitively eliminate this virus from the blood supply. If transfusion-transmitted JEV is confirmed to be a significant threat to the blood supply in highly endemic regions, a combination of these methods might be required to prevent transfusion-transmitted JEV infection.

Transmission of WNV, another member of the JEV antigenic serocomplex, to immunocompromised patients via blood transfusion has been reported (*7*). Immunocompromised patients infected with WNV tended to have longer incubation periods (>10 days) and higher rates of severe illness (*39*). In our study, the index patient had symptom onset 14 days after the transfusion and overt encephalitis 2 days later, a relatively long incubation period, comparable with the observations made for WNV. Clinical specimens from immunocompetent patients with JEV infection are typically PCR negative at symptom onset, reflecting immune-mediated pathogenesis of the disease. However, in our immunocompromised patient, JEV RNA was detected in a serum specimen from 10 days before symptom onset and in bronchoalveolar fluid and CSF samples from days 5 and 6, respectively, after symptom onset, reflecting the inability of this immunocompromised patient to effectively clear the virus. The detection of JEV in lower respiratory tract specimens has not been reported previously. However, WNV has been reported to cause pneumonia in immunocompromised transplant recipients (*40*). The clinical significance of JEV in the respiratory tract of our patient is unclear; the patient had extensive consolidative changes over both lung fields on chest radiograph, but culture of the bronchoalveolar fluid specimen yielded *B. cepacia*, suggesting a component of bacterial pneumonia. However, pulmonary involvement caused by disseminated JEV infection in this immunocompromised patient cannot be excluded.

We identified a second patient who had evidence of JEV seroconversion after receiving platelets from the viremic donor. This patient was immunocompromised at the time of platelet transfusion, which was 19 days after completion of induction chemotherapy for acute leukemia. His relatively asymptomatic clinical course was probably related to the recovery of bone marrow function during the incubation period, in contrast to the index patient, who was subjected to continuous lymphocyte-depleting immunosuppression from the antirejection prophylaxis administered during the incubation period. However, other factors might be responsible, including differences in virus inoculum (the JEV envelope protein has hemagglutinating properties, which could have resulted in a higher accumulation of virus in the packed red blood cell packet); blood product storage conditions (platelet concentrates are stored at higher temperatures, which could have lowered JEV viability in this blood product); and other subtle differences in host-pathogen interactions.

In summary, this study illustrates that JEV can be transmitted via transfusion of cellular blood components to immunocompromised persons and could cause severe outcomes. Enhanced understanding of the prevalence of JEV in the blood supply, the incidence of transfusion-transmitted JEV, and measures for risk mitigation in JEV-endemic areas are urgently needed.

Technical Appendix 1 Description of real-time reverse transcription PCR for detection of Japanese encephalitis virus.

Technical Appendix 2 Questionnaire given by Hong Kong Red Cross to blood donors.
