# Supplementary material for: Japanese Encephalitis Virus Transmitted Via Blood Transfusion, Hong Kong, China
Source: Emerg Infect Dis. 2018 Jan;24(1):49–57. doi: 10.3201/eid2401.171297 (PMC5749455; doi:10.3201/eid2401.171297)
Supplement: Technical Appendix 1 — Description of real-time reverse transcription PCR for detection of Japanese encephalitis virus. [file 17-1297-Techapp-s1.pdf]

Article DOI: http://dx.doi.org/10.3201/eid2401.171297

# Japanese Encephalitis Virus Transmitted Via Blood Transfusion, Hong Kong, China

## Technical Appendix

### One-Step Real-Time Reverse Transcription PCR

To detect Japanese encephalitis virus (JEV), we used RealTime ready RNA Virus Master kit (Roche, Indianapolis, Indiana, USA) and real-time PCR machine LC2.0 (Roche). We used the following primers and probe: JE-F(e) (5′-GGAGCTGGATGGAATGTGAA-3′, 10 µM); JE-R(e) (5′-TCCCTCCGATGGAAGTATAGAA-3′, 10 µM); and JE-P(e) (6-FAM-CCAAAGCGTATGCACAGATGTGGC-BBQ-650, 5 µM). Equine herpes virus primers, probes, and DNA were also included in reactions as a DNA internal control. The thermocycler conditions for the real-time reverse transcription PCR (RT-PCR) were as follows: reverse transcription at 50ºC for 15 min; heat inactivation at 95ºC for 30 s; 45 cycles of denaturation at 95ºC for 1 s, annealing at 55ºC for 20 s, and extension at 72ºC for 5 s; and cooling at 40ºC for 30 s.

The limit of detection of our assay (using a –7 dilution of cell culture extract, cycle threshold ($C_t$) 36.10–37.50; using a –8 dilution of cell culture extract, $C_t$ 42.60 to undetectable) is comparable with that of the 1-step conventional nested RT-PCR for detection of flaviviruses when tested by using a –8 serial dilution of JEV RNA extracted from a mosquito isolate grown in C6/36 cell culture. Cross reactivity to dengue virus serotypes 1, 2, 3, and 4; chikungunya virus; and West Nile virus was not detected. Archived samples previously positive for JEV (3

mosquito isolates and 2 clinical specimens [brain tissue and cerebrospinal fluid]) were all positive by our in-house assay, and 51 JEV-negative archived RNA samples taken from cerebrospinal fluid (3 herpes simplex virus 2–positive, 3 varicella zoster virus–positive, and 45 negative) were retested and still negative by our in-house test. The 1-step real time RT-PCR with internal control has comparable limit of detection as the 1-step conventional nested RT-PCR for detection of flaviviruses and was adopted for routine use in 2014.
