# Supplementary material for: Japanese Encephalitis Virus Transmitted Via Blood Transfusion, Hong Kong, China
Source: Emerg Infect Dis. 2018 Jan;24(1):49–57. doi: 10.3201/eid2401.171297 (PMC5749455; doi:10.3201/eid2401.171297)
Supplement: Technical Appendix 2 — Questionnaire given by Hong Kong Red Cross to blood donors. [file 17-1297-Techapp-s2.pdf]

Article DOI: http://dx.doi.org/10.3201/eid2401.171297

Japanese Encephalitis Virus Transmitted Via Blood Transfusion, Hong Kong, China

Technical Appendix

The following pages show questionnaire given by Hong Kong Red Cross to blood donors.

DIN

HONG KONG RED CROSS
BLOOD TRANSFUSION SERVICE

BLOOD DONATION REGISTRATION FORM

PART I: PRE-DONATION INFORMATION

Thank you for coming to give blood today. Your donation could save and change the lives of the recipients. We sincerely request you to read our blood donation information precisely. **To protect your safety in giving blood and the safety of the recipients of your donation, it is vital that we review your suitability to donate today.** If you are uncertain about any questions in this form or in need of more blood donation information, please talk to our nurse on duty.

After donation, your blood will be stringently tested, inter alia, blood groups and infectious diseases, before processed into blood products. Donations that meet all the quality and safety standards will be issued for patient use in Hong Kong. However, some will be selected for quality assurance testing, academic or medical research. In addition, it may be made available to patients outside Hong Kong for humanitarian considerations or if there is a genuine surplus to local needs.

Giving blood is not completely risk-free as adverse reactions may occasionally happen. These include bruising, pain, inflammation, infection or skin allergy around the needle puncture site, dizziness or fainting after donation. They usually are mild and short-lasting. In the event of adverse reactions, our nurse will provide on-site care and arrange referral to hospital, if necessary. We sincerely request you to read and follow our "Post-Donation Advice".

We would use your contact information (name, address, telephone and email) provided in this form to keep you informed of blood donation activities. In addition, if your blood is tested positive for any of the infection, we shall inform you accordingly. Should you have any queries, please feel free to ask our nurse on duty.

For Reference Only

SAFE BLOOD SAVE LIVES

NOT ALL BLOOD BORNE INFECTIONS CAN BE DETECTED BY LABORATORY TESTS. PLEASE HELP US ENSURE BLOOD SAFETY AND DO NOT PROCEED TO DONATE IF YOU SUSPECT THAT YOUR BLOOD MAY CARRY A POTENTIAL RISK OF INFECTION OR IF YOU WANT TO HAVE YOUR BLOOD TESTED.

FOR FREE HIV TESTING, PLEASE CONSULT YOUR DOCTOR OR CALL 2780 2211.

If you find this blood donation registration form, please contact our staff at 2710 1333.

WIBCS201F1_19

PART II: HEALTH SCREENING AND INFECTION RISK ASSESSMENT

You are required to answer the following questions honestly by putting a "✓" in the correct box. If you do not understand any questions in this form or not certain how to respond, please leave the question blank and seek clarification from our nursing staff later. Our nurse will review your responses, answer your questions and discuss with you IN CONFIDENCE to determine if you can donate today. The information you disclose will be kept in strict confidence. Thank you.

**General Health Screening**	**YES**	**NO**	**Staff Use**
A1. Are you feeling well enough to give blood today?			
A2. Are you currently under a doctor's treatment, taking any medication (including herbal medicine) or awaiting test result?			
A3. Have you ever been diagnosed of the following illnesses?			
• cardiovascular diseases (e.g. chest pain, hypertension)			
• respiratory diseases (e.g. asthma)			
• gastrointestinal or liver diseases (e.g. inflammatory bowel disease, hepatitis)			
• blood diseases (e.g. bleeding problem)			
• cancers			
• endocrine or metabolic diseases (e.g. diabetes, thyroid diseases)			
• neurological diseases (e.g. loss of consciousness, epilepsy)			
• mental disorders			
• kidney or urogenital diseases (e.g. nephritis, kidney or bladder stones)			
• autoimmune or rheumatological diseases (e.g. SLE, rheumatoid arthritis)			
A4. Have you ever been diagnosed of G6PD deficiency?			
A5. Have you ever taken the following drugs?			
• aspirin or any drugs containing aspirin			
• non-steroidal anti-inflammatory drugs			
• drugs for hair loss			
• drugs for benign prostatic hypertrophy			
• drugs for acne			
A6. Have you ever had drug allergy? If yes, please specify:____________________			
A7. **If you are male**, please continue the questionnaire on the next page.			
**If you are female**,			
• Are you pregnant?			
• Have you given birth/ had an abortion in the last 12 months?			
• Are you still breast-feeding?			
• Have you ever received treatment for infertility?			

For Reference Only

WIBCS201F1_19

**HIV/ AIDS, Hepatitis B and Hepatitis C Infection Risk** *Assessment (Some of the questions below are related to your sexual life. Sexual contact in this Form refers to oral, vaginal or anal sex, with or without the use of condom.)*	**YES**	**NO**	**Staff Use**
B1. Have you been diagnosed of or suspected to have the following?			
• HIV infection/ AIDS			
• Hepatitis B infection			
• Hepatitis C infection			
B2. Have you ever had sex for money?			
B3. Have you ever used or injected yourself with narcotics or non-prescribed medication?			
B4. Have you ever received clotting factor concentrates?			
B5. Are you aware of your sexual partner(s)			
• has been diagnosed of HIV infection/ AIDS?			
• has ever been a sex worker?			
• has ever used or injected narcotics or non-prescribed medication?			
• has ever received clotting factor concentrates?			
B6. • **If you are male**, have you ever had oral or anal sex with a man?			
• **If you are female**, have you ever had sexual contact with a bisexual man (one who has had oral or anal sex with another man)?			

**CJD and vCJD (Mad-cow Disease) Infection Risk Assessment**	**YES**	**NO**	**Staff Use**
C1. **Between 1 January 1980 and 31 December 1996,**			
• have you spent a total of three or more months in the UK?			
• have you worked or lived for a total of six or more months at US Military bases in Europe?			
C2. **Between 1 January 1980 and the present,**			
• have you spent a total of five or more years in Europe?			
• have you received blood transfusion in the UK or France?			
• have you received bovine insulin injection?			
C3. Have you ever received			
• pituitary derived human growth hormone or human gonadotrophin?			
• organ or tissue transplant?			
C4. Have any of your blood relatives been diagnosed of Creutzfeldt-Jakob Disease (CJD)?			

For Reference Only

Other Recent Infection or Vaccination Risk Assessment	YES	NO	Staff Use
D1. **In the past 1 week**, have you had any dental procedure (including scaling, dental extraction, etc.), open wounds or skin lesions?			
D2. **In the past 2 weeks**, have you had symptoms of flu, fever, headache,eye pain, muscle or joint pain, vomiting, enlarged lymph nodes or skin rash?			
D3. **In the past 4 weeks,**			
• have you had contact with someone with an infectious disease e.g. chickenpox, rubella, tuberculosis (TB)?			
• have you had any vaccinations e.g. vaccination against Hepatitis A, Hepatitis B or tetanus?			
• have you had diarrhea?			
D4. **In the past 12 months,**			
• have you travelled outside Hong Kong?<br>Destination(s):____________________________<br>Date of return to HK (DD/MM/YY):________________			
• have you had tattoo, acupuncture, ear or body piercing, or accidental needle stick injury?			
• have you been given Hepatitis B Immune Globulin?			
• have you been bitten by any animal?			
• have you undergone surgical operation (including endoscopic examination, treatment involving the use of catheters)?			
• have you received blood transfusion?			
D5. Have you been diagnosed of the following infectious diseases?			
• Malaria			
• Venereal disease			
• Tuberculosis (TB)			
• Glandular fever			
• SARS			
• Dengue Fever			
• West Nile Virus Infection			
• Chikungunya			
• Others, please specify:________________________			

For Reference Only

Other Risk Factors Assessment	YES	NO	Staff Use
E1. Have you ever donated blood under another name?			
E2. Have you ever been informed not to donate blood permanently by us or other blood service?			
E3. Have you resided outside Hong Kong consecutively for 6 months or longer during the past three years?<br>If yes, your previous country of residence:__________________			
E4. Will you be undertaking any hazardous sport today?<br>e.g. rock climbing, diving or flying			
E5. Will you be driving a heavy vehicle or working at hazardous depths or heights today? e.g. fireman, train or lorry driver, or scaffolding worker			

Supplementary Question	YES	NO	Staff Use
F1. Are you aware of your sexual partner(s)			
• Has been diagnosed of Zika infection?			
• Has been resided or returned from the Zika virus affected areas in the past 3 months?			

PART III: DECLARATION

I solemnly and sincerely declare that I have read, understood and agreed with **'Part I: PRE-DONATION INFORMATION'** and the staff on duty has answered all my queries.

I solemnly and sincerely declare that all information which I have provided in **'Part II: HEALTH SCREENING AND INFECTION RISK ASSESSMENT'** is true. I also consent to have my blood tested for infectious diseases (including HIV) by the Hong Kong Red Cross Blood Transfusion Service and to be informed if my blood is tested positive.

Donor Signature: ____________________ Date: ____________________ Verified by Screening nurse Signature: ______________
(Please sign in front of screening nurse)

For Reference Only

WIBCS201F1_19

PART IV : PERSONAL INFORMATION

(Corresponds to Personal Identity Document) (Remark: Photocopy of form is not accepted)
If there is no change of contact details, previous donors are required to fill in items with * asterisk only.

DIN

*Name ______________________
Surname Other Name

Name in Chinese ______________ (if applicable)
Telegraph code (______)(______)(______)(______) (if applicable)

*HK ID. No. ______________ (   )
*Date of Birth (DD) (MM) (YY)
*Sex ________

*Weight ________ (kg)
*Height ________ (cm)
Blood Group ________ (if known)
Donor ID. ________

Have you donated blood in HK Yes ☐ No ☐
*Last Donation Date ________

Corresponding Address ______________________
(Please provide accurate address for future correspondence) ______________________
H.K. ☐ KLN. ☐ N.T. ☐

Daytime Tel. No. ________ Nighttime Tel. No. ________

*Mobile No. ________

Email Address ________

Donor Label

☐ I do not wish to receive information relating to the publicity and promotion of blood donation activities.

For Reference Only

For Office Use Only
(Please ✓ where appropriate)

Blood Pack Lot No.

Drive ID : ________

Donor Examination

Hb Test Performed By ________ BP ________ mmHg P ________ /min.

Hb/Counter Reading ____/____ g/dL Temp ________ ^{o}C
(Highlight heading when out of range)

Hb/Counter Eq. No. EIHMC: EIACA:

Time Hb tested __ __ : __ __ hr.:min.

Apheresis

Plasma only ☐
Plasma & Platelet ☐
Platelet only ☐

Remarks

Require new card ☐
Aspirin ☐
Stain/Mark ☐
Small Vein ☐
Autologous Donation ☐
Directed Donation ☐

Lignocaine

Yes ☐ No ☐

Ferrous Sulphate ☐
300mg/tablet
(14 tablets for 14 days)
One tablet daily

Fortifer FA ☐
300mg/tablet
(16 tablets for 16 days)
One tablet daily

Dispensed By ________

Staff Code ________

Blood Unit Weigher Equipment No. : EIMIX /EIBAL /EICPP

Hand Held Sealer Equipment No. : EITCS

No. of DIN Labels Used / Destroyed : ________ / ________

Blood Flow

Start Time __ __ : __ __ hr.:min.
End Time __ __ : __ __ hr.:min.
Duration ________ min.

Blood Pack (WB Donation)

Quadruple 450 ☐
Quadruple 350 ☐
Single ☐

Volume Collected

Over Collection____ml ☐
Low Volume Unit____ml ☐
Inadequate Collection____ml ☐
Fail____ml ☐

Health Screened By :	Staff Code	Access for Venepuncture:	Lt / Rt (1st)	Lt / Rt (2nd)		(1st)	(2nd)
Special Message :		Accepted for Donation and Venepuncture By:			Staff Code		
		Venepuncture Completed By:			Staff Code		
☐ Deferral ☐ Hold ☐ Remark Code :		Deferral Duration :					
Comment :					Staff Code		

WIBCS201F1_19
